# Do host species evolve a specific response to slave-making ants?

**DOI:** 10.1186/1742-9994-9-38

**Published:** 2012-12-31

**Authors:** Olivier Delattre, Rumsaïs Blatrix, Nicolas Châline, Stéphane Chameron, Anne Fédou, Chloé Leroy, Pierre Jaisson

**Affiliations:** 1 Laboratoire d’Ethologie Expérimentale et Comparée, Université Paris 13, Sorbonne Paris Cité, 99 avenue J.-B, Clément, 93430, Villetaneuse, France; 2 Centre d’Ecologie Fonctionnelle et Evolutive, Centre National de la Recherche Scientifique, 1919 route de Mende, F-34293, Montpellier cedex 5, France; 3 Departamento de Biologia, FFCLRP, Universidade de São Paulo (USP), Ribeirão Preto, SP, Brazil

**Keywords:** Coevolution, Formicidae, Social recognition, Social parasitism, Temnothorax

## Abstract

**Background:**

Social parasitism is an important selective pressure for social insect species. It is particularly the case for the hosts of dulotic (so called slave-making) ants, which pillage the brood of host colonies to increase the worker force of their own colony. Such raids can have an important impact on the fitness of the host nest. An arms race which can lead to geographic variation in host defenses is thus expected between hosts and parasites. In this study we tested whether the presence of a social parasite (the dulotic ant *Myrmoxenus ravouxi*) within an ant community correlated with a specific behavioral defense strategy of local host or non-host populations of *Temnothorax* ants. Social recognition often leads to more or less pronounced agonistic interactions between non-nestmates ants. Here, we monitored agonistic behaviors to assess whether ants discriminate social parasites from other ants. It is now well-known that ants essentially rely on cuticular hydrocarbons to discriminate nestmates from aliens. If host species have evolved a specific recognition mechanism for their parasite, we hypothesize that the differences in behavioral responses would not be fully explained simply by quantitative dissimilarity in cuticular hydrocarbon profiles, but should also involve a qualitative response due to the detection of particular compounds. We scaled the behavioral results according to the quantitative chemical distance between host and parasite colonies to test this hypothesis.

**Results:**

Cuticular hydrocarbon profiles were distinct between species, but host species did not show a clearly higher aggression rate towards the parasite than toward non-parasite intruders, unless the degree of response was scaled by the chemical distance between intruders and recipient colonies. By doing so, we show that workers of the host and of a non-host species in the parasitized site displayed more agonistic behaviors (bites and ejections) towards parasite than toward non-parasite intruders.

**Conclusions:**

We used two different analyses of our behavioral data (standardized with the chemical distance between colonies or not) to test our hypothesis. Standardized data show behavioral differences which could indicate qualitative and specific parasite recognition. We finally stress the importance of considering the whole set of potentially interacting species to understand the coevolution between social parasites and their hosts.

## Introduction

Parasitism is an interspecific interaction where the host species suffers from the exploitation of its resources by the parasite. Any defense strategy minimizing the impact of the parasite on host fitness is therefore likely to be selected for. In this case, escalation of reciprocal counter-adaptations between the parasite and its host is likely to lead to an "arms race" [1]. This coevolutionary process is considered an important factor that shapes species life history and plays a key role in the geographic mosaic of coevolution through local adaptations [2,3]. Thus, elucidating patterns of local host-parasite interactions helps understanding prominent factors in the evolution of life history traits.

Social parasitism is a complex parasite-host interaction since it not only involves individuals but societies. Mainly found in social bees, wasps and ants, social parasites use the worker force of another social species to rear their own brood [4]. In the case of dulotic species (so called slave-makers), the parasite workers are specialized for conducting raids in a two-step process [5]. First, scouts individually search for potential host nests. When successful, the scout returns to its nest and recruits nestmates to initiate the raid, during which slave-maker ants seize brood and bring it back home. Later, host workers emerging in the parasite nest will be imprinted on and integrated into the mixed colony where they rear the parasite brood, feed and groom the parasite workers, defend the nest against aliens, and even participate in raids. Raids can jeopardize host colony survival [6-8], therefore exerting a strong selection pressure upon the hosts. Reciprocally, there is some evidence that hosts also exert a selection pressure on their parasites in return [9], since resistance by host colonies might prevent enslavement [10]. Coevolutionary processes between dulotic ant species and their hosts then can escalate to an evolutionary arms race [11,12].

In ants, social recognition is based on cuticular hydrocarbon profiles which display qualitative differences between distinct species and quantitative ones between homospecific colonies [13-15]. Since each species is characterized by a qualitatively unique blend of cuticular hydrocarbons [14], an innate mechanism to detect some chemical cues from the parasite could have evolved in host species. In addition, we know that ants can learn to recognize aliens and modify their subsequent behavior in a variety of social contexts [16-18] and social parasites have been proven to modify host behavior in some species [12,19,20].

*Myrmoxenus ravouxi* is a dulotic social parasite with a wide distribution in Europe ranging from France to Greece [21]. It is known to parasitize several species of the diversified genus *Temnothorax*[21-23]. Some *Temnothorax* species are potential hosts for several social parasites [5], while others are never parasitized. Interestingly, the distribution range of the hosts often exceeds the parasite’s, leaving some host populations parasite-free. This ant genus thus allows for comparative analysis of defense strategy both between species (host and non-host) and within species (populations with and without parasites). In this study we tested whether the presence of a social parasite within an ant community correlated with a specific behavioral defense strategy of local host or non-host populations. Behavioral responses of colonies to the introduction of parasite (*M. ravouxi*), homospecific and heterospecific workers were compared between hosts (*Temnothorax unifasciatus* and *T. rabaudi*) and non-host (*T. nylanderi*) species from two sites, one with and one without the parasite. We expected the behavioral response of host species’ colonies in the parasitized population to be more aggressive toward parasite intruders than toward other non-parasite intruders.

It is usually assumed that agonistic interactions between ants depend on the dissimilarity between cuticular hydrocarbon profiles [14,24,25], with aggressiveness escalating when dissimilarity increases [26,27]. A new theoretical model recently emerged based on empirical data [28], which suggests that ants may discriminate aliens using the presence of undesirable chemical compounds absent from their nestmates’ chemical signature [25], therefore focusing on qualitative differences rather than on global dissimilarity. To separate out quantitative and qualitative components of the chemical recognition, we assessed the chemical quantitative dissimilarity between colonies by calculating the Euclidean distance using chromatogram peaks areas [29-31] for components that were found in all groups, leaving out components that occurred only in some groups and were therefore responsible for qualitative differences between cuticular profiles (see supplementary data). Such methodology amounts to calculating the distance between colonies in a n-dimensional space where all peaks have the same weight (1/n). This simple calculation is not intended to mirror the actual sensory and cognitive treatment used by ants in social decision-making processes, but it provides us with a way to quantitatively assess chemical distance without referring to any special role for particular compounds. If host species have evolved a specific recognition mechanism toward their parasite, the behavioral response to *M. ravouxi* should not be fully explained simply by overall cuticular profile dissimilarity, but also involve qualitative detection of some specific compounds. The agonistic response of *Temnothorax* host species, or populations, to the social parasite should therefore fulfill two criterions to be considered specific: a) it should be different from the response to other non-parasite species, and b) it should be at least partially independent of the global dissimilarity between chemical profiles.

## Results

### Cuticular hydrocarbon profiles

**Figure 1:**
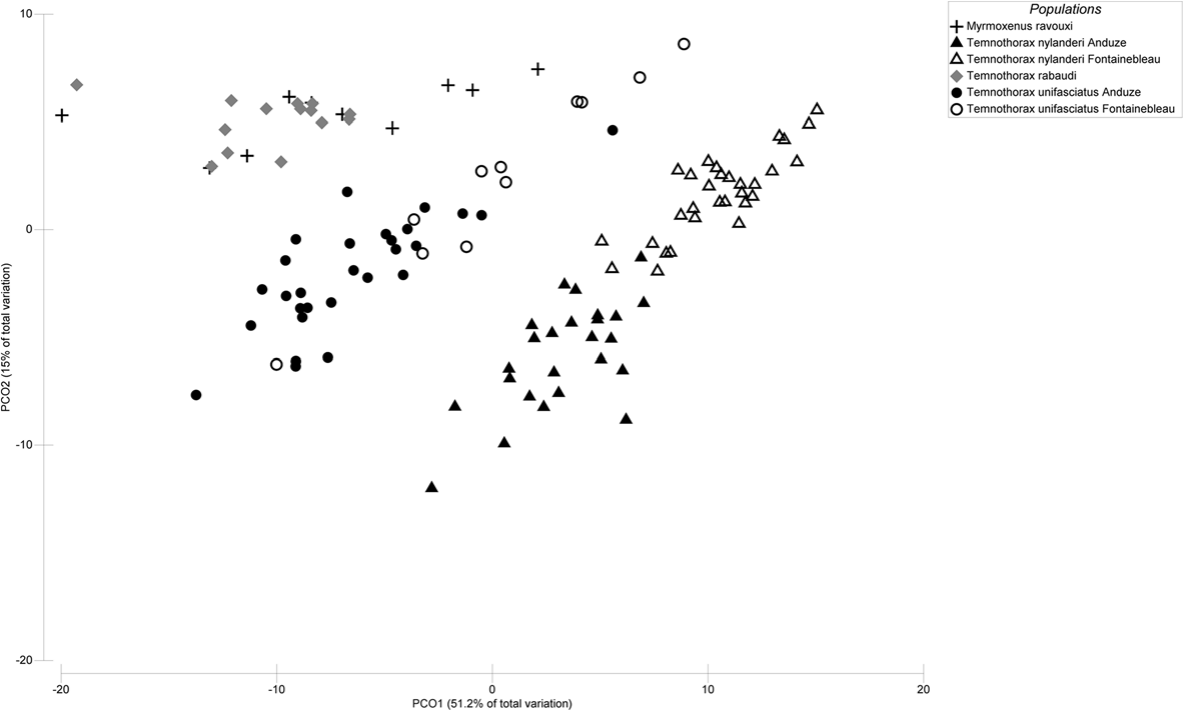
**Principal coordinate analysis.** Principal coordinate analysis (PCO) based on the resemblance matrix calculated from Euclidean distances between every pair of samples for the 41 peaks present in all populations of *M. ravouxi*, *T. unifasciatus*, *T. nylanderi* and *T. rabaudi* from Anduze (myr_an, uni_an, nyl_an and rab_an respectively), and of *T. unifasciatus* and *T. nylanderi* from Fontainebleau (uni_f and nyl_f respectively).

The principal coordinate analysis (PCO, Figure 1) clearly sorts out our different populations (cf. Additional file 1). Cuticular hydrocarbon profiles of the colonies of the different populations were significantly different overall (PERMANOVA, pseudo-F = 42.931 *P* < 0.001), and in all pairwise comparisons (PERMANOVA, Pair-wise tests, all *P* < 0.006). A canonical analysis (data not shown) shows that more than 99.2% (117/118) of our samples were correctly assigned to their respective populations, the only exception being a *T. unifasciatus* colony from Fontainebleau assigned to the Anduze population of the same species. As expected, *M. ravouxi* profiles were close to those of *T. rabaudi* slaves (see mean chemical distances in Additional file 1), but still different, which is consistent with both theoretical and empirical data on mixed species colonies [27,32-34].

### Agonistic response of the recipient colonies

#### Bites

**Figure 2:**
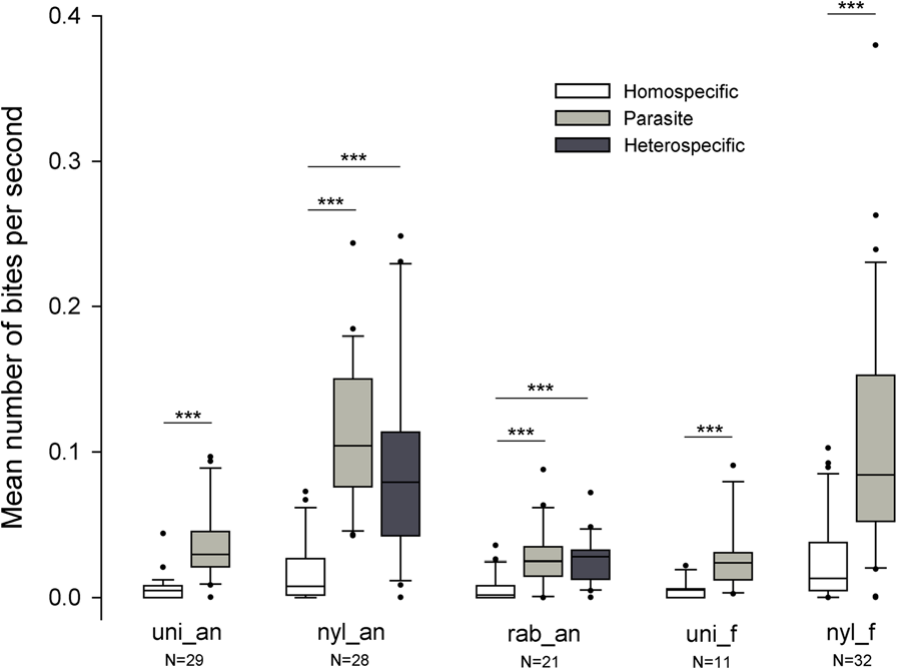
**Box-and-whisker plots for bite rates inflicted in recipient colonies.** Introductions were carried out in colonies from populations of *M. ravouxi*, *T. unifasciatus*, *T. nylanderi* and *T. rabaudi* from Anduze (myr_an, uni_an, nyl_an and rab_an respectively), and of *T. unifasciatus* and *T. nylanderi* from Fontainebleau (uni_f and nyl_f respectively). Box plots show the median and 25-75% percentiles. Whiskers show all data excluding outliers outside the 10th and 90th percentiles (circles). Figures under bars indicate number of tests. *** *P* < 0.001.

For each population, colonies were more aggressive toward parasites than toward homospecifics (Figure 2; paired permutation tests, *P’* < 0.001).

The Kruskal-Wallis analysis showed a significant effect of the “population” factor on the response to introduced homospecific workers (Figure 2; H = 17.69, *P* = 0.0014). The non-host species (*T. nylanderi*) was more aggressive in Fontainebleau than in Anduze (permutation test, *P’* = 0.034), while other pairwise comparisons were not significant (permutation tests, all *P’* > 0.089).

The “population” factor proved also significant for the colony responses to introduced parasite workers (Figure 2; Kruskal-Wallis, H = 55.77, *P* < 0.001). The non-host species was significantly more aggressive than the other species at both sites (permutation tests, in Anduze, compared with *T. unifasciatus*: *P’* < 0.001; *T. rabaudi*: *P’* < 0.001; in Fontainebleau, *T. unifasciatus*: *P’* = 0.007). *T. nylanderi* colonies from Anduze were more aggressive toward parasite workers than *T. nylanderi* colonies from Fontainebleau (permutation test, *P’* = 0.0056).

**Figure 3:**
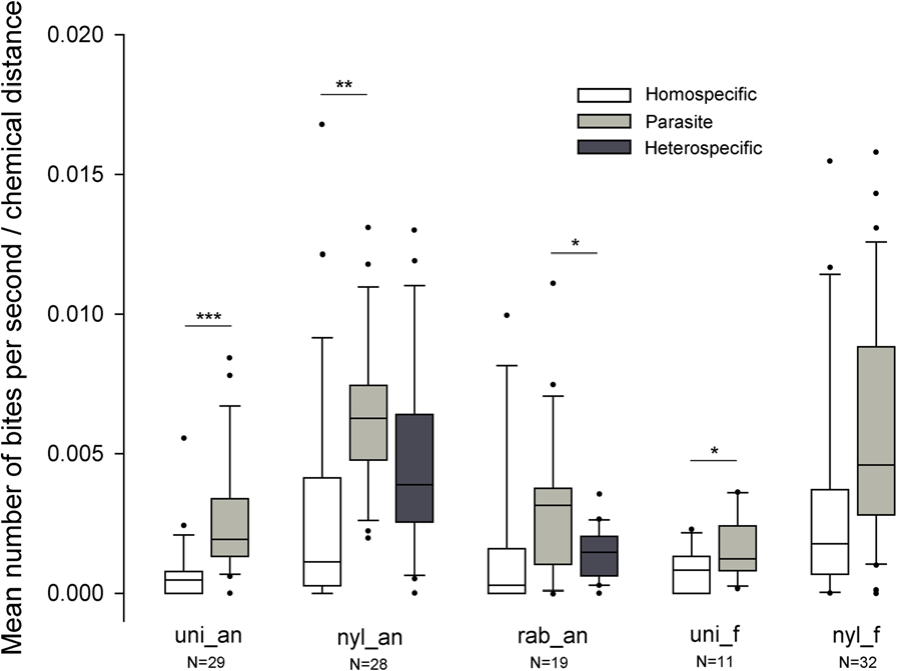
**Box-and-whisker plots for bite rates inflicted in recipient colonies scaled by chemical distance.** We scaled the mean bite rates by chemical distance between the intruders and the recipient colonies from populations of *M. ravouxi*, *T. unifasciatus*, *T. nylanderi* and *T. rabaudi* from Anduze (myr_an, uni_an, nyl_an and rab_an respectively), and of *T. unifasciatus* and *T. nylanderi* from Fontainebleau (uni_f and nyl_f respectively). Box plots show the median and 25-75% percentiles. Whiskers show all data excluding outliers outliers outside the 10th and 90th percentiles (circles). Figures under bars indicate number of tests. * *P* < 0.05, ** *P* < 0.01, *** *P* < 0.001.

Introduction of a non-parasite heterospecific worker was performed in colonies of *T. nylanderi* and *T. rabaudi* from the parasitized site (Anduze) in order to test whether ants were able to discriminate the parasite from another heterospecific worker. For both species, the respon-ses toward parasites, homospecifics and heterospecifics were significantly different (Figure 3; Kruskal-Wallis, *T. nylanderi*: H = 44, *P* < 0.001; *T. rabaudi*: H = 19.02, *P* < 0.001). For both species the agonistic response was lowest toward homospecific intruders (Figure 3; permutation tests, *P’* < 0.001) but was not significantly different toward parasites or heterospecific non-parasites (permutation tests, *P’* = 0.108 and *P’* = 0.577, respectively).

### Bites (scaled by chemical distance)

For each population except *T. rabaudi* from Anduze and *T. nylanderi* from Fontainebleau (Figure 3; paired permutation tests, *P’* = 0.342 and *P* = 0.15), colonies were significantly more aggressive toward parasites than toward homospecifics (Figure 3; paired permutation tests, all *P* < 0.011).

Colony responses to introduced homospecific workers significantly differed between populations (Figure 3; Kruskal-Wallis, H = 17.88, *P* = 0.0013). The non-host species (*T. nylanderi*) was more aggressive than *T. unifasciatus* in Anduze (Permutation test, *P’* = 0.028) but not in Fontainebleau (*P’* = 0.317). We found no differences between sites for either *T. unifasciatus* or *T. nylanderi* (*P’* = 1 and *P’* = 1, respectively).

Colony responses to introduced parasite workers also differed significantly between populations (Figure 3; Kruskal-Wallis, H = 35.5, *P* < 0.001). The non-host species was significantly more aggressive than the other species in both sites (permutation tests, in Anduze, compared with *T. unifasciatus*: *P’* < 0.001; *T. rabaudi*: *P’* = 0.0016; in Fontainebleau, *T. unifasciatus*: *P’* = 0.022). The response of non-host against parasites was not significantly different between sites (permutation test, *P’* = 1.1268).

Introduction of a non-parasite heterospecific worker was performed in colonies of *T. nylanderi* and *T. rabaudi* from the parasitized site (Anduze). For both species, the responses toward parasites, homospecifics and heterospecifics were significantly different (Figure 3; Kruskal-Wallis, *T. nylanderi*: H = 18.71, *P* < 0.001; *T. rabaudi*: H = 9.082, *P* = 0.011). *T. nylanderi* colonies were marginally more aggressive toward parasites than toward heterospecific non-parasites (Figure 3; permutation test, *P’* = 0.086). No statistical difference could be observed for the agonistic response of *T. rabaudi* between homospecific heterocolonial intruders and heterospecific ones, be they parasites or not (all *P’* > 0.342). However, *T. rabaudi* were significantly more aggressive toward parasites than toward heterospecific non-parasite intruders (permutation test, *P’* = 0.018).

### Ejections

**Figure 4:**
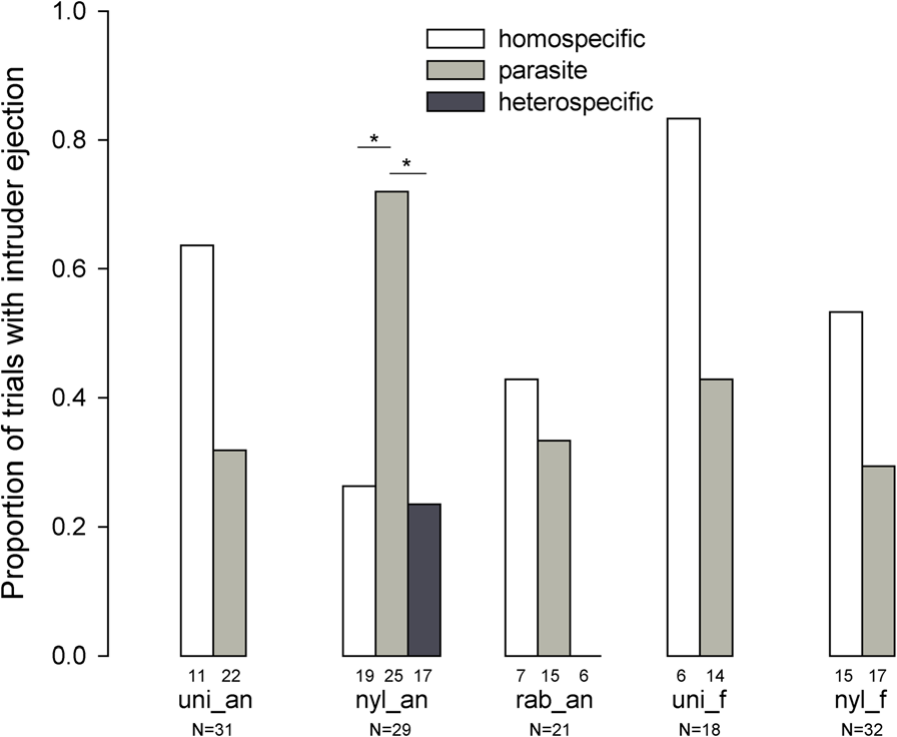
**Proportion of trials with intruder ejection.** Proportion of trials with intruder ejection when they did not escape the nest (figures under bars indicate number of tests without escape, N indicate total number of tests) for populations of *M. ravouxi*, *T. unifasciatus*, *T. nylanderi* and *T. rabaudi* from Anduze (myr_an, uni_an, nyl_an and rab_an respectively), and of *T. unifasciatus* and *T. nylanderi* from Fontainebleau (uni_f and nyl_f respectively). * *P’* < 0.05.

Ejection rates of homospecific intruders were not significantly different between populations (Figure 4, Fisher-Freeman-Halton test, *P’* = 0.095). On the contrary, ejection rates of parasites were significantly different between populations (Figure 4, Fisher-Freeman-Halton test, *P’* = 0.002): the non-host species (*T. nylanderi*) from the parasitized site (Anduze) tended to display this behavior more often than other populations (Fisher tests, comparison with *T. unifasciatus* from Anduze: *P’* = 0.052; *T. rabaudi* from Anduze: *P’* = 0.094; *T. nylanderi* from Fontainebleau: *P’* = 0.057) but it was not significant when compared to *T. unifasciatus* from Fontainebleau (*P’* = 0.381). More importantly, *T. nylanderi* from Anduze was the only population in which parasites were ejected significantly more often than homospecific intruders (ejection rates respectively 0.72 and 0.26, Fisher test, *P’* = 0.011); it also ejected parasite workers significantly more frequently than heterospecifics (ejection rates respectively 0.72 and 0.24, Fisher test, *P’* = 0.012), whose ejection rate did not differ from homospecifics’ (Fisher test, *P’* = 1).

## Discussion

In Anduze, all four species could be discriminated using their cuticular hydrocarbon profiles. Profiles of *M. ravouxi* were very close to those of the main host, *T. rabaudi*, suggesting integration of host components into the chemical profile of the parasite. This mechanism has already been shown in dulotic ants [35-40]. However, in our study, the profiles of the parasite and its main host were distinct (cf. Additional file 1). GC-MS analyses also showed some qualitative differences between the parasite and its host (cf. Additional file 2). Chemical analyses thus prove the potentiality for hosts to recognize the parasite and therefore to display a specific defense behavior, even if the chemical closeness of the social parasite might also make recognition more difficult.

With the raw data, all studied populations reacted more aggressively to allospecific intruders (be they parasites or not) than to homospecific heterocolonial ones. The non-host species *T. nylanderi*, both from the parasitized (Anduze) and non-parasitized site (Fontainebleau), was more aggressive toward parasite intruders than the other species.

Neither *T. rabaudi* nor *T. nylanderi* from Anduze reacted more aggressively to the parasite than to a non-parasite heterospecific intruder. Response of *T. unifasciatus* to the parasite was not significantly different between Anduze and Fontainebleau (the parasite-free site). Thus, we could not detect any clear-cut effect of the presence of the parasite in the ant community on the defense strategy of the two host species.

This pattern could be explained by the fact that, in this host-parasite local interaction, the parasite species is leading the arms race and offsets any specific defense through mimetic odor [35,41] or propaganda allomone [42]. Moreover, as opportunist *M. ravouxi* slave-making ants can use more than five host species [23,43], it may shift from one host species to another in the same site, for example as a consequence of any counter adaptations from its host species. An alternative hypothesis would be that the cost of being parasitized is lower than the cost of a specific defense which could increase nestmate recognition errors [44] and decrease colony fitness.

The raw results show that host and non-host species are not specifically more aggressive toward parasite than non parasite intruders, but the non-host species display more aggressiveness toward these intruders than the host species. *T. nylanderi* populations are sometimes very dense [45,46] and competition for nest sites may cause colony fusion. These conflicts and subsequent intraspecific parasitism may select for a higher level of aggressiveness in dense populations of this species. In Fontainebleau, where we found 1–3 colonies per square meter, *T. nylanderi* display a higher degree of aggressiveness toward homospecific intruders. We thus may hypothesize that in this site, *T. nylanderi* colonies lowered their tolerance threshold in response to intraspecific competition.

Interestingly, *T. nylanderi* colonies from the parasitized site of Anduze bit more often parasite intruders than *T. nylanderi* colonies from the non-parasitized site of Fontainebleau. However, the difference between the medians is very small (0.104 vs. 0.0842), and thus we cannot conclusively interpret them as a product of local adaptation processes.

In order to investigate a potential specific response to the slave-making parasite *M. ravouxi* parasite, which could not be fully explained by the global chemical dissimilarity, we also compared agonistic responses when scaled according to the quantitative chemical distance between host and intruder colonies. A slightly different picture arises from this second analysis. Differences between homospecific and parasite introductions remain significant, with the exception of *T. rabaudi* colonies from Anduze and *T. nylanderi* from Fontainebleau, which are not significantly more aggressive toward parasite than toward homospecific intruders.

*T. rabaudi* host colonies from the parasitized population (Anduze) were more aggressive toward parasite than non-parasite heterospecific workers. *T. nylanderi* colonies displayed a stronger agonistic response while biting more often parasite than homospecific intruders and showing a tendency to be more aggressive toward parasite than non-parasite heterospecific intruders after standardization (*P’*= 0.086). Host species colonies did not show more aggressiveness toward non-parasite heterospecifics than toward homospecifics intruders in our second analysis. Parasite intruders were extracted from *T. rabaudi* parasitized colonies and were thus more likely to bear some of the specific chemical cues this host species displays. It could explain why *T. rabaudi* colonies did not show a higher aggressiveness toward the parasite workers when we scaled down the importance of quantitative differences. Still, free-living host colonies did show a higher agonistic response to parasite than to non-parasite heterospecific intruders. We thus suggest that the more aggressive response to parasite than to heterocolonial homospecifics or non-parasite heterospecifics after standardization indicates that ant aggressiveness does not solely increase linearly with global chemical distance. Pamminger and collaborators [20] showed that aggressive reaction of *Temnothorax longispinosus* colonies against non-parasite congeneric workers transiently rose after the introduction of a dead slave-making *Protomognatus americanus* worker, when the introduction of a dead congeneric worker had no visible consequence. They concluded that *T. longispinosus* specifically reacted to the parasite presence in an adaptive way by lowering their general tolerance threshold, since *Temnothorax* enslaved workers join parasite workers during raids and fiercely attack host colonies. Parasite presence indeed reliably signals a forthcoming raid, which is not the case for congeneric workers that can belong to non-parasite colonies. We thus may hypothesize that *Temnothorax* ants rely on qualitative analysis of the cuticular profiles to discriminate parasite from non-parasite species in parasitized populations. This could result from ants weighting more some compounds (be they specific to the parasite or not), either because of the cognitive processes used to match the perceived cuticular profile and the internal template [14,44], or because sensory systems are more sensitive to qualitative differences than quantitative ones (some heterospecific compounds are absent of the internal template, while homospecific profiles simply differ in a quantitative way) [26], or both, since these mechanisms are not mutually exclusive.

In some behavioral tests, recipient workers also ejected the intruder. Such a behavior is probably not very efficient in preventing raids in host colonies, because parasite workers could easily return to the raided nest. However, it could have been selected as a ritualized fighting behavior [47-49] in non-host species since it allows conflict resolution without casualties (the intruder usually put up little resistance). The ejection strategy could thus have been selected to prevent fights in cases where a social parasite scout enters a colony from the non-host colony. *M. ravouxi* scouts also have no interest in engaging in fights in non-host species colonies. Slave-making ants may discriminate host from non-host species colonies, using for example a recognition template based on innate or experience-induced preferences [50,51]. They could then also actively avoid conflicts with non-host workers by inducing ejection behaviors. Nonetheless, in order to limit the interaction effect on ejections by recipient colonies, we use proportion of ejections only where the intruders did not escape. Moreover, *T. nylanderi* from the parasitized site of Anduze showed a higher rate of ejection of parasite intruders than of non-parasite heterospecifics or heterocolonial homospecifics, while ejection rates displayed by *T. nylanderi* in the parasite-free site (Fontainebleau) were not different between parasite and homospecific intruders. Besides, *T. nylanderi* ejected the parasite more often in Anduze than in Fontainebleau. Taken together, these results show that *T. nylanderi* from the parasitized site displayed a specific response to the social parasite, even though it is not used as a host. The specific response consisted in a higher rate of a ritualized behavior and marginally more agonistic interactions (respectively ejections and bites). *T. nylanderi* has never been observed as host, but our results suggest potential interactions between the two species. This is obviously difficult to verify because raids are rarely observed in the field. Thus, we cannot know if the specific behavior has been locally selected per se, or if it results from the learning of parasite odor through experience prior to colony collection. To tell apart the respective roles of local selection and learning capacities, similar experiments should be conducted with *T. nylanderi* colonies reared in the lab from founding queens from Anduze.

## Conclusions

Our results did not show unequivocal evidence of a host behavioral defense that would be specific to the social parasite *M. ravouxi.* Nonetheless, standardization of agonistic behaviors by chemical distance between intruder and receiver colonial signatures shows a higher aggressiveness toward the parasite than toward a non-parasite heterospecific intruder for the host species, which could result from a coevolutionary arms race [7,8,10,11,37]. We do not know how ants’ decision rules are implemented, i.e. following qualitative or quantitative dissimilarity of chemical cues. Ants certainly may adjust their behavior according to the absence/presence of some particular chemical cues when facing conspecifics [51,52]. So, we think that standardization of behavioral results by chemical distance between colonies in social insects may bring new insights on the discrimination mechanisms towards sympatric species of social parasites. In this aspect, our methodology shows a pattern of host resistance. Moreover, we show for the first time that a species never recorded as host (*T. nylanderi*) can also display a specific behavioral defense to a slave-making ant. The specific defense included higher rates of bites and ritualized ejections of parasite workers. It remains to be tested whether *M. ravouxi* attempts raids on *T. nylanderi* in the field. Our results lead us to stress the importance of considering the whole set of potentially interacting species to fully understand the geographic mosaic of host-parasite interactions [53,54].

## Materials and methods

### Study species

The parasite species *M. ravouxi* was collected in Anduze (N44°3’ E3°59’). *T. unifasciatus* and *T. rabaudi* are common hosts of *M. ravouxi*. *T. unifasciatus* colonies were collected both in the Anduze site and in Fontainebleau (N48°24’ E2°42’), which is outside the distribution area of *M. ravouxi*. *T. rabaudi* could only be found in Anduze, where it is the most frequent host for *M. ravouxi* (some parasite colonies where found with *T. unifasciatus* slave workers). Last, we collected *T. nylanderi* colonies in both the Anduze and Fontainebleau sites. To the best of our knowledge, *T. nylanderi* has never been recorded as a host for any social parasite (the records of Bernard [55,56] could not be confirmed by Buschinger et al. [57]); however, it could be found in Anduze in sympatry with the slave-making *M. ravouxi*. In Fontainebleau, *T. nylanderi* was very frequent, with densities of almost 2 or 3 colonies per square meter. This could limit the number of available nest sites and cause intraspecific colony fusions and parasitism in this species [58].

Throughout the rest of this manuscript refer to "populations" for colonies of a given species originating from the same site: *T. unifasciatus* from Anduze (uni_an, N = 29), *T. unifasciatus* from Fontainebleau (uni_f, N = 11), *T. nylanderi* from Anduze (nyl_an, N = 28), *T. nylanderi* from Fontainebleau (nyl_f, N = 32), *T. rabaudi* from Anduze (rab_an, N = 21), and *M. ravouxi* from Anduze (myr_an, N = 13). The latter were only used as a source of parasite workers to introduce in the free-living tested colonies. These parasite workers were thus all extracted from *T. rabaudi* parasitized colonies. We also refer to *T. nylanderi* as “non-host species”, *T. unifasciatus* and *T. rabaudi* as “host species”, Fontainebleau as “non-parasitized site” and Anduze as “parasitized site”.

Colonies were all queen-right and reared in the lab in artificial nests made of two microscope slides superimposed and kept 1 mm apart from each other using a thin piece of linoleum. They were fed with honey and fruit flies. The experiments took place in the spring, from April to May, after a three-month wintering period at 8°C. The colonies were collected at the end of the previous summer. The temperature during the experiments was around 24°C.

### Characterizing dissimilarity between cuticular hydrocarbons profiles

The cuticular hydrocarbons profile was characterized for each colony by soaking 10 workers in 50 μl of pentane for ten minutes. An aliquot of 2 μl was analyzed using gas-chromatography with flame ionization detection (GC-FID) on a Varian 3900 instrument equipped with a split/splitless injector and a DB5 fused silica capillary column (30 m x 0.32 mm, 0.25 μm film thickness), with helium as carrier gas at a flow rate of 28.57 cc/sec. The temperature was maintained at 100°C for 5 min, raised at 3°C/min to 300°C and held constant for 10 min. Peak areas were calculated using the Varian system control. Peaks were later identified using a GC-MS, allowing us to exclude contaminants from analyses. Analyses were carried out on a Agilent 5975 C inert XL with chromatograph GC system 7890A of Agilent with a split-splitless injector and a fused-silica capillary column (30 meters long with a diameter of 250 μm) with a 0.25 μm polydimethylsiloxane coating. The carrier gas was helium (99.99%) and the column temperature program was 100°C (5 min) and 3°/min to 300°C (10 min). The injection port temperature was 280°C. Total ion chromatograms and mass spectra were recorded in the electron impact ionization mode at 70 eV. The transfer line and the source temperature were maintained at 230°C. Compound identifications were based on retention times and comparison with published data [59]. In a first step, peaks displaying an area above or equal to 1% of the total area in at least 10 samples (66 peaks) were selected. In a second step, we discarded all peaks that were not present in all populations, to focus on quantitative rather than qualitative differences between chemical profiles; this lead to a final selection of 41 peaks. Euclidean distances between every pair of samples were calculated to produce a resemblance matrix. A principal coordinate analysis (PCO) using this matrix was then performed to sort out the cuticular hydrocarbon profiles of the colonies. We compared the colonial profiles of our populations with a single factor PERMANOVA [60,61] using 9999 permutations, first to detect overall differences and then in one-by-one tests between the populations. Statistical analyses on the chemical profiles were carried out with the PERMANOVA+ V1.0.2 add-on package [62] of PRIMER V6.1.12 [63]. The mean distance between populations’ centroids was also calculated to illustrate the calculated quantitative similarity between them (Additional file 1).

### Behavioral observations

The tests consisted in the introduction of an alien worker inside a colony, slightly removing the upper side of the nest to obtain a small opening on one side of the colony and to introduce the intruder directly inside it. All colonies were tested twice, using a parasite (*M. ravouxi*) and a conspecific heterocolonial intruder (from the same site). *M. ravouxi* alien workers came from parasitized colonies of *T. rabaudi* (N = 13). For *T. rabaudi* and *T. nylanderi* from Anduze, a third test was performed using a heterospecific, non-parasite intruder (*T. rabaudi* for *T. nylanderi* and vice versa, both from the same site and from free-living colonies). The sequence of introductions (parasite, heterocolonial and heterospecific non-parasite) was randomized between colonies to avoid any order effect. We used only local conspecifics or heterospecifics for introductions, except for *M. ravouxi* intruders, as this species could only be found in the south of France. We did so because coevolutionary processes, which could lead to pattern of specific resistance, are obviously likely to appear locally [2].

The agonistic response of the recipient colony was assessed by recording bites and ejection of the intruder by resident ants (see definitions below) until the alien worker left the nest, and in the few cases this did not happen within 30 minutes the test was terminated. Test duration was thus variable, but lower than 30 minutes.

### Bites

We compared the agonistic response of colonies during the tests using the bite rates (number of bites divided by test duration).

We also performed a second analysis of our agonistic response by dividing these rates by the chemical distance between the intruder and the recipient colony, to test for a specific recognition on qualitative chemical cues. The rationale here is that a specific recognition mechanism would lead to higher levels of agonistic interactions towards the parasite than towards other intruders, even after getting rid of their global chemical distance with the recipient colony.

The chemical distance D was defined as the Euclidean distance between chemical profiles [30-32]:

$$D\left(x,y\right)=\sqrt{\sum_{i}{\left(\mathrm{xi}-\mathrm{yi}\right)}^{2}}$$

where x and y refer to the two colonies, x_*i*_ and y_*i*_ to the relative areas of peak *i* for colonies x and y respectively. When the chemical profile of a colony was not available (for technical reasons), we used the mean chemical profile of the corresponding population. This was the case for 5 homospecific tests with *T. nylanderi* from Fontainebleau, 11 homospecific tests with *T. unifasciatus* from Fontainebleau, 14 homospecific and 7 non-parasite heterospecific tests with *T. rabaudi* from Anduze. In our second analysis, we had to discard two *T. rabaudi* colonies confronted in a test (recipient/intruder) because we did not know their chemical profile.

Comparisons were performed using Kruskal-Wallis and permutation tests with general scores for paired samples [64]. We compared populations from different species within the same site and populations from the same species between sites. Other comparisons were considered irrelevant, i.e. different species from different sites. In order to use paired permutation tests between the two (or three) behavioral tests for each population (using the sequential Bonferroni-Holm correction procedure when necessary [65]), the few recipient colonies that had performed only one type of test were discarded from the analysis (respectively 7 and 2 for *T. unifasciatus* from Fontainebleau and Anduze, and 1 for *T. nylanderi* from Anduze).

### Ejections

Apart from bites we also observed, in some trials, recipient ants grasping the intruder by an appendage and dragging it outside of the nest, a behavior we referred to as “ejection”. Test outcomes could thus be of three types: the intruder escaped, it was ejected by residents or it could stay in the nest for the whole observation period. To assess the resident propensity to eject intruders, we calculated the proportion of ejections over the tests where the intruder did not escape.

All comparisons were done using Fisher or Fisher-Freeman-Halton tests [66]. We used the sequential Bonferroni-Holm correction procedure [65] when necessary, and adjusted p-values are noted *P’* (they should be compared to the standard 0.05 significance threshold) [67].

All statistical tests were performed with StatXact (Cytel Studio, version 8.0.0, 2007).

## Abbreviations

Myr: an stands for *Myrmoxenus ravouxi* from Anduze; nyl: an for *Temnothorax nylanderi* from Anduze site; nyl: f for *Temnothorax nylanderi* from Fontainebleau; rab: an for T*emnothorax rabaudi* from Anduze; uni: an for T*emnothorax unifasciatus* from Anduze and uni_f for T*emnothorax unifasciatus* from Fontainebleau.

## Competing interests

The author(s) declare that they have no competing interests.

## Authors’ contributions

OD participated in statistical, chromatography and behavioral analysis and wrote the manuscript. RB conceived the study, performed the GC analysis, participated in statistical, chromatography and behavioral analysis and helped to draft the manuscript. NC participated in the GC-MS and behavioral analysis and helped to draft the manuscript. SC performed the GC analysis, participated in statistical, chromatography and behavioral analysis and helped to draft the manuscript. AF carried out the behavioral assays. CL carried out the GC-MS analysis and helped to identify chemical compounds. PJ participated in the design and coordination of the study. All authors read and approved the final manuscript.

## Supplementary Material

Additional file 1 **Distances between centroids.** Distance between centroids for the chemical profiles of populations of *M. ravouxi*, *T. nylanderi* from Anduze and Fontainebleau, *T. rabaudi* and *T. unifasciatus* from Anduze and Fontainebleau. Click here for file

Additional file 2 **Identified cuticular hydrocarbons of species’ chemical profiles.** Mean percentages (±S.D) of the different compounds in the chemical profiles of *M. ravouxi (M. rav)*, *T. unifasciatus* from Anduze (*T. uni_And*) and Fontainebleau *(T. uni_Font), T. rabaudi (T. rab)* and *T. nylanderi* from Anduze (*T. nyl_And*) and Fontainebleau (*T. nyl_Font*). Compounds were identified using GC-MS. Unidentified hydrocarbons are marked with *. Click here for file
