# Supplementary material for: Do host species evolve a specific response to slave-making ants?
Source: Front Zool. 2012 Dec 31;9:38. doi: 10.1186/1742-9994-9-38 (PMC3551654; doi:10.1186/1742-9994-9-38)
Supplement: Additional file 1 — Distances between centroids. Distance between centroids for the chemical profiles of populations of M. ravouxi, T. nylanderi from Anduze and Fontainebleau, T. rabaudi and T. unifasciatus from Anduze and Fontainebleau. [file 1742-9994-9-38-S1.doc]

| **Distance between centroids** | *M. ravouxi* | *T. nylanderi*  Anduze | *T. nylanderi*  Fontainebleau | *T. rabaudi*  Anduze | *T.unifasciatus*  Anduze |
| --- | --- | --- | --- | --- | --- |
| *M. ravouxi* |  |  |  |  |  |
| *T. nylanderi*  Anduze | 16.25 |  |  |  |  |
| *T. nylanderi*  Fontainebleau | 19.145 | 12.528 |  |  |  |
| *T. rabaudi*  Anduze | 6.723 | 17.985 | 21.916 |  |  |
| *T. unifasciatus*  Anduze | 10.266 | 12.764 | 18.169 | 11.503 |  |
| *T. unifasciatus*  Fontainebleau | 12.161 | 11.712 | 13.452 | 15.236 | 10.096 |

Additional File 1. Distance between centroids of the chemical profiles
